# *Pithovirus sibericum*, a new *bona fide* member of the “Fourth TRUC” club

**DOI:** 10.3389/fmicb.2015.00722

**Published:** 2015-08-04

**Authors:** Vikas Sharma, Philippe Colson, Olivier Chabrol, Pierre Pontarotti, Didier Raoult

**Affiliations:** ^1^Unité de Recherche sur les Maladies Infectieuses et Tropicales Emergentes, UM63, Centre National de la Recherche Scientifique 7278, IRD 198, Institut National de la Santé et de la Recherche Médicale U1095, Aix-Marseille UniversityMarseille, France; ^2^I2M UMR 7373, Centre National de la Recherche Scientifique Équipe Evolution Biologique et Modélisation, Aix-Marseille UniversityMarseille, France; ^3^Institut Hospitalo-Universitaire (IHU) Méditerranée Infection, Pôle des Maladies Infectieuses et Tropicales Clinique et Biologique, Fédération de Bactériologie-Hygiène-Virologie, Centre Hospitalo-Universitaire Timone, Assistance Publique-Hôpitaux de MarseilleMarseille, France

**Keywords:** giant virus, *Pithovirus sibericum*, *Megavirales*, nucleocytoplasmic large DNA viruses, informational genes, TRUC, domains of life, phylogeny

## Abstract

Nucleocytoplasmic large DNA viruses, or representatives of the proposed order *Megavirales*, include giant viruses of *Acanthamoeba* that were discovered over the last 12 years and are *bona fide* microbes. Phylogenies based on a few genes conserved amongst these megaviruses and shared by microbes classified as *Eukarya, Bacteria*, and *Archaea*, allowed for delineation of a fourth monophylogenetic group or “TRUC” (Things Resisting Uncompleted Classification) composed of the *Megavirales* representatives. A new *Megavirales* member named *Pithovirus sibericum* was isolated from a >30,000-year-old dated Siberian permafrost sample. This virion is as large as recently described pandoraviruses but has a genome that is approximately three to four times shorter. Our objective was to update the classification of *P. sibericum* as a new member of the “Fourth TRUC” club. Phylogenetic trees were constructed based on four conserved ancient genes and a phyletic analysis was concurrently conducted based on the presence/absence patterns of a set of informational genes from members of *Megavirales, Bacteria, Archaea*, and *Eukarya*. Phylogenetic analyses based on the four conserved genes revealed that *P. sibericum* is part of the fourth TRUC composed of *Megavirales* members, and is closely related to the families *Marseilleviridae and Ascoviridae/Iridoviridae*. Additionally, hierarchical clustering delineated four branches, and showed that *P. sibericum* is part of this fourth TRUC. Overall, phylogenetic and phyletic analyses using informational genes clearly indicate that *P. sibericum* is a new *bona fide* member of the “Fourth TRUC” club composed of representatives of *Megavirales*, alongside *Bacteria, Archaea*, and *Eukarya*.

## Introduction

Nucleocytoplasmic large DNA viruses (NCLDVs), or members of the proposed order *Megavirales*, are the largest known viruses thus far; their genome ranges in size from ≈100 to 2500 kilobase pairs (kbp) and they are visible on photonic microscopy (Iyer et al., 2006; Raoult and Forterre, 2008; Yutin et al., 2009; Yutin and Koonin, 2012; Colson et al., 2013; Philippe et al., 2013; Legendre et al., 2014; Raoult, 2014). These viruses rely on very diverse eukaryotic hosts for their life cycle, which include protists, algae, vertebrate animals, and insects. They include giant viruses whose existence was revealed 12 years ago with the discovery of Mimivirus, a *Acanthamoeba polyphaga*-resisting microbe that was initially considered a Gram positive bacterium (La Scola et al., 2003; Raoult et al., 2004) and then discovered to have a gigantic 1200 kbp-large genome encompassing approximately 1000 genes (Raoult et al., 2007; Raoult, 2014). Moreover, this genome was predicted to encode several cellular trademark genes never previously reported in any kind of viruses. Afterwards, several dozen new giant amoebal viruses were discovered that were almost all isolated on *Acanthamoeba* spp., including other mimiviruses (Fischer et al., 2010; Arslan et al., 2011; Yoosuf et al., 2012), but also smaller giants that founded the family *Marseilleviridae* (Aherfi et al., 2014), then pandoraviruses, which are the current record holders for virion and genome size (Philippe et al., 2013; Antwerpen et al., 2015). These *Megavirales* members were shown to share a putative ancient common ancestor that was inferred to harbor about 50 core genes (Koonin and Yutin, 2010; Yutin and Koonin, 2012). Still more recently, other researchers described another giant virus, *Pithovirus sibericum*, which also infects *Acanthamoeba* and was isolated in Siberia from a >30,000-year-old frozen soil sample (Legendre et al., 2014). The *P. sibericum* virion has a very similar morphology and size compared to pandoraviruses but its genome was unexpectedly about 3–4 times shorter than those of pandoraviruses, and comparative genomics showed low similarity between these new clades of giant viruses. In addition, only 11 and 32% of the *P. sibericum* gene complement were found to have counterparts in other viruses and the NCBI GenBank sequence database, respectively. The greatest numbers of best matches with viruses were found to be with representatives from the *Marseilleviridae*, then *Mimiviridae* and *Iridoviridae* families. It is noteworthy that only a very weak homology with a major capsid protein from an unclassified megalocytivirus of the family *Iridoviridae* was found in the *P. sibericum* gene content.

Cellular organisms were divided in the 1970s by C. Woese into three domains (*Bacteria, Archaea*, and *Eukarya*) comprising a universal tree of life on the basis of ribosomal genes (Woese et al., 1990). This way, viruses were neglected and excluded from this tree of life as devoid of ribosomes (Raoult, 2013, 2014). After the giant virus discovery, this classification was challenged because a few conserved genes from these viruses appeared to share a common ancestor with homologs from cellular organisms. Thus, starting from the initial description of the Mimivirus genome, several phylogeny reconstructions and phyletic analyses based on such genes showed that *Megavirales* comprised a fourth major monophyletic group alongside *Bacteria, Archaea*, and *Eukarya* in a revised universal tree of life (Boyer et al., 2010; Raoult, 2014; Sharma et al., 2014, 2015). The four branches topology was criticized by other groups that claim it was biased by long branch attraction or lateral transfer of genes to giant viruses from their hosts or other sources (Filee et al., 2008; Moreira and Brochier-Armanet, 2008; Williams et al., 2011; Yutin et al., 2014). However, alternative trees failed to show a monophyly for *Eukarya* (Williams et al., 2011). In addition, although giant viruses have mosaic genomes that harbor genes from multiple origins and with a complex evolutionary history (Filee et al., 2008; Boyer et al., 2009; Yutin and Koonin, 2012), there are a few genes not prone to transfer (Sharma et al., 2014). Using two of them, which are universal and encode DNA-dependent RNA polymerase subunits, recent phylogenetic analyses with a comprehensive sequence set further supported the four branches of life hypothesis (Sharma et al., 2014). In addition, other research groups strengthened this hypothesis using different methodological approaches or datasets (Wu et al., 2011; Nasir et al., 2012). Eventually, the term “TRUC” (an acronym for Things Resisting Uncompleted Classifications) was introduced for a new classification of life that includes a fourth TRUC, consisting of *Megavirales*, standing out from the ribosome-based three domain classification (Raoult, 2013, 2014), and we recently described pandoraviruses as new members of a “Fourth TRUC” club (Sharma et al., 2015). Here, we tested the membership of the new and highly divergent amoebal giant virus *P. sibericum* to this “Fourth TRUC” club.

## Materials and methods

### Orthologous sequences from viruses

Analysis was performed using a strategy similar to the one we have used in previous works (Boyer et al., 2010; Sharma et al., 2014) to update the fourth TRUC hypothesis. The genes used in the present study were identified from clusters of orthologous groups of proteins (COGs) involved in nucleotide transport and metabolism and information storage and processing (i.e., categories F, J, A, K, L, and B). These genes included five genes conserved among previously identified *Megavirales* representatives and that encode family B DNA polymerase (DNApol), DNA-dependent RNA polymerase subunits 1 (RNAP1) and 2 (RNAP2), transcription factor II B (TFIIB), and ATP-dependent DNA ligase (DNA ligase). All these genes have an important function in replication and transcription processes (Yutin and Koonin, 2009; Boyer et al., 2010). DNApol plays a critical role in replication, thus transferring genetic information from one generation to another; it is one of the most widely used ancient gene markers for taxonomic mapping across a wide range of organisms from *Archaea, Eukarya*, and *Megavirales*, but was found less conserved among *Bacteria* (Filee et al., 2002). Genes encoding RNAP subunits are considered as an appropriate alternative to ribosomal genes because they are conserved in *Bacteria, Archaea, Eukarya*, and *Megavirales*, and poorly prone to horizontal gene transfer and recombination (Case et al., 2007; Adekambi et al., 2009; Sharma et al., 2014). TFIIB is a transcriptional regulator that plays an important role in transcription and was found to be conserved in a few *Megavirales* representatives, among which *P. sibericum* is found, as well as in a wide range of cellular organisms among *Eukarya* and *Archaea*. Finally, ATP-dependent DNA ligase is involved in the process of replication, repair, and recombination, and is widely conserved in *Bacteria, Eukarya*, phages, and some *Megavirales* representatives (including phycodnaviruses, chordopoxviruses, asfarviruses, marseilleviruses, and *P. sibericum*) (Yutin and Koonin, 2009). Viral orthologs for these four genes were extracted using the OrthoMCL program (Li et al., 2003) from the gene complements of a set of 317 viral genomes harboring more than 100 genes, which were directly downloaded from the NCBI sequence databases (ftp://ftp.ncbi.nih.gov/genomes/Viruses/) and were completed with the gene complement previously determined from the *P. sibericum* (GenBank Accession no. NC_023423.1).

### Orthologous sequences from the cellular life forms

Stand-alone BLAST 2.2.27 searches were performed to retrieve homologs for informational genes from the members of cellular domains of life, using viral sequences as a query against the NCBI GenBank non-redundant (nr) protein sequence database; the maximum number of target sequences was 20,000 (Altschul et al., 1990). Because informational genes are the most conserved genes among all life forms, they comprise large amounts of homologous sequences in public databases. To obtain an informative equilibrated phylogenetic tree, we selected homologous sequences using TimeTree, a professional informational data bank where the divergence times of species have been reported based on studies of molecular clocks from peer-reviewed journals (Hedges et al., 2006). Here, we have selected representative species that have been diverging since around 500 million years ago to constitute a set of representatives from *Bacteria, Archaea*, and *Eukarya* (Sharma et al., 2014). A Perl script was used to filter out from the BLASTp results, by Taxon identifiers, sequences from these selected representatives. Then, selected protein sequences were directly downloaded from the NCBI nr database using the GenBank identifiers. Identical sequences were removed by clustering using the CD-HIT suite, as previously described (Sharma et al., 2014, 2015).

### Multiple sequence alignments and phylogeny reconstructions

Sequences were aligned by the Muscle program (Edgar, 2004). Alignment quality was analyzed visually and manually curated, and phylogeny reconstructions were performed with the maximum likelihood method and the WAG model; confidence values were calculated by the Shimodaira-Hasegawa (SH) test using FastTree (Price et al., 2010). The FigTree software was used for the visualization of phylogenetic trees (http://tree.bio.ed.ac.uk/software/figtree/).

### Phyletic pattern analysis with COGs

DNA processing and nucleotide metabolism genes are highly conserved in all cellular organisms and in some viruses (Wolf et al., 2002; Boyer et al., 2010). In addition, clusters of orthologous groups of proteins (COGs) have been documented as an ideal set to study functional annotations (Tatusov et al., 2003). Therefore, a set of 727 COGs involved in information storage, processing, nucleotide transport, and metabolism (categories J, A, K, L, B, and F) have been used for the current analysis. Stand-alone BLASTp was performed for the corresponding 727 COGs against selected representatives from *Bacteria, Archaea, Eukarya*, and *Megavirales*, with stringent parameters (*e* < 1e-3, query coverage >70%, and identity >30%) (Boyer et al., 2010). Then, a binary matrix was constructed using BLASTp results based on patterns of presence (1) and absence (0). Finally, a dendrogram was constructed by hierarchical clustering amended with the Pearson distance method using the TM4 multi-package software (Saeed et al., 2003).

## Results

### Phylogenetic analyses

**Figure 1:**
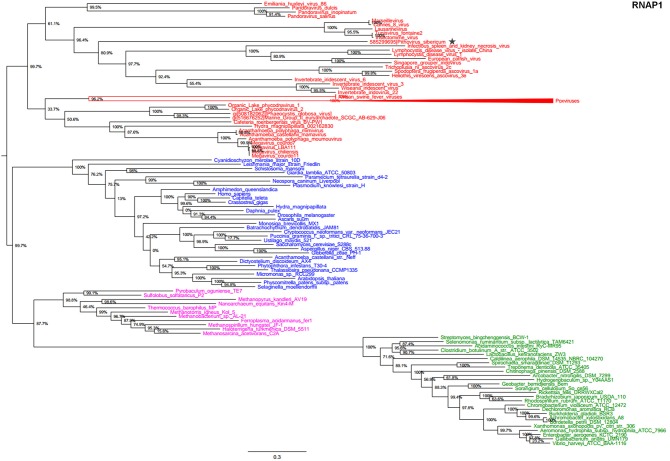
**RNAP1 phylogenetic tree**. The RNAP1 tree was built by using aligned protein sequences from *Megavirales* (red), *Bacteria* (green), *Archaea* (pink), and *Eukarya* (blue). Confidence values were calculated by the Shimodaira-Hasegawa (SH) test using the FastTree program (Price et al., 2010). Average length of sequences was 1345 amino acids. The scale bar represents the number of estimated changes per position. The star indicates *Pithovirus sibericum*.

**Figure 2:**
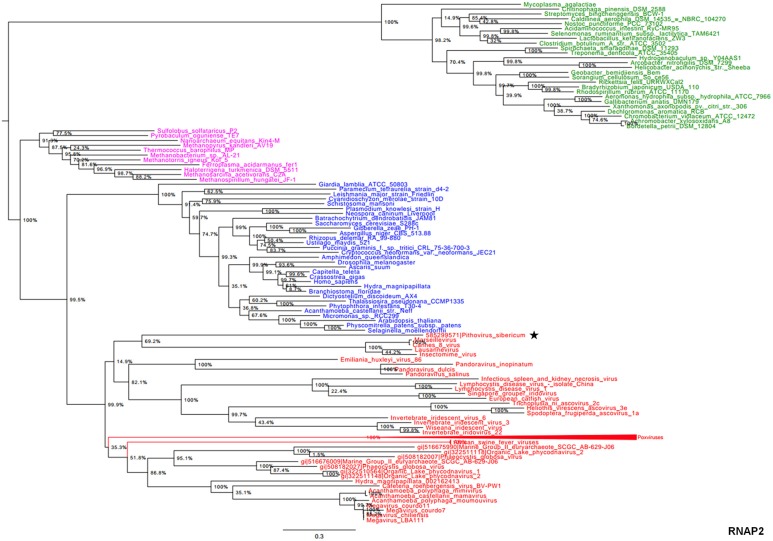
**RNAP2 phylogenetic tree**. The RNAP2 tree was built by using aligned protein sequences from *Megavirales* (red), *Bacteria* (green), *Archaea* (pink), and *Eukarya* (blue). Confidence values were calculated by the SH test using the FastTree program (Price et al., 2010). Average length of sequences was 1195 amino acids. The scale bar represents the number of estimated changes per position. The star indicates *Pithovirus sibericum*.

In the present study, we reconstructed phylogenies using five informational genes. *P. sibericum* position in both RNAP1 and RNAP2 trees indicated that this species belongs to a new family of *Megavirales*, and was most closely related to families *Iridoviridae/Ascoviridae* and *Marseilleviridae* (Figures 1, 2). In the RNAP1 tree, *P. sibericum* was clustered with iridoviruses and ascoviruses, whereas marseilleviruses appeared as an outgroup. In the RNAP2 tree, *P. sibericum* was confidently clustered with marseilleviruses. In addition, these phylogenetic trees constructed using both RNAP1 and 2 sequences from representatives of *Bacteria, Archaea, Eukarya*, and *Megavirales*, including pandoraviruses and *P. sibericum*, clearly delineated four branches.

**Figure 3:**
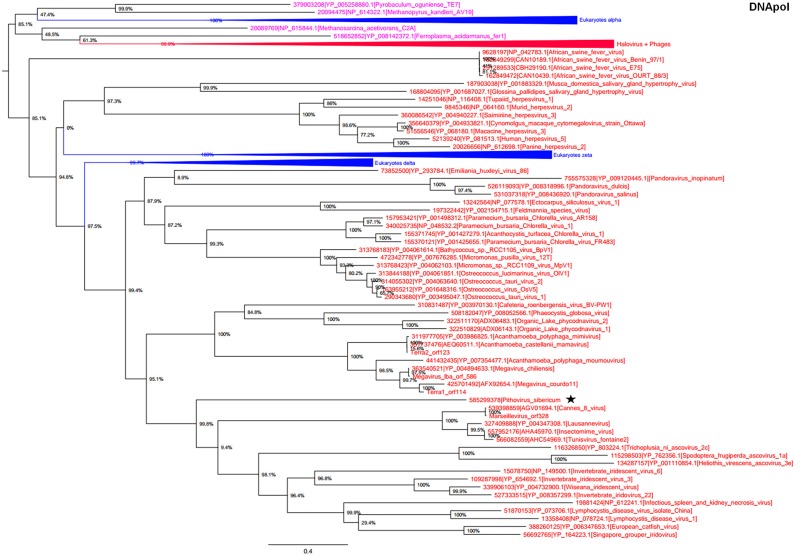
**DNA polymerase phylogenetic tree**. The DNA polymerase tree was built by using aligned protein sequences from *Megavirales* (red), *Bacteria* (green), *Archaea* (pink), and *Eukarya* (blue). Confidence values were calculated by the SH support using the FastTree program (Price et al., 2010). Average length of sequences was 1190 amino acids. The scale bar represents the number of estimated changes per position. The star indicates *Pithovirus sibericum*.

In the phylogeny reconstruction based on DNApol that is present in archaea, eukaryotes, and megaviruses, *P. sibericum* branched deeply in a cluster that also included marseilleviruses, iridoviruses, and ascoviruses (Figure 3). In addition, all these viruses were clustered together with other *Megavirales* members, apart from asfarviruses.

**Figure 4:**
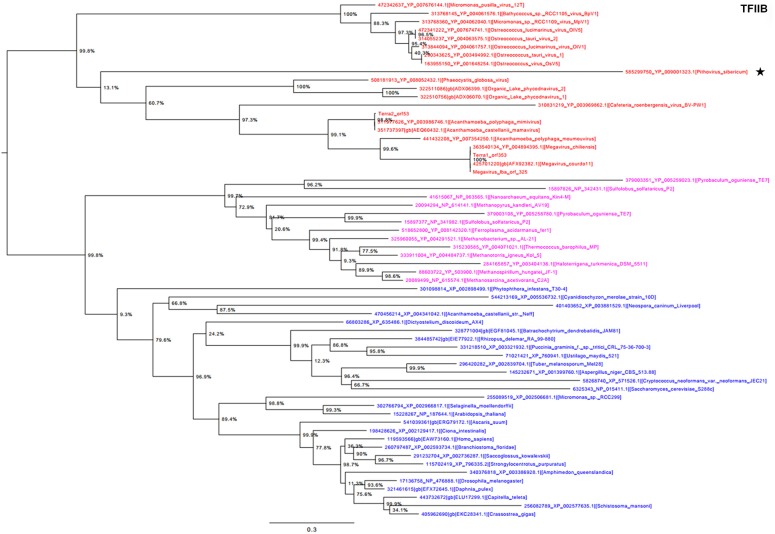
**Transcription factor II B (TFIIB) phylogenetic tree**. The TFIIB tree was built by using aligned protein sequences from *Megavirales* (red), *Bacteria* (green), *Archaea* (pink), and *Eukarya* (blue). Confidence values were calculated by the SH support using the FastTree program (Price et al., 2010). Average length of sequences was 348 amino acids. The scale bar represents the number of estimated changes per position. The star indicates *Pithovirus sibericum*.

Phylogeny reconstructed using TFIIB showed a strongly supported monophylogenetic group composed of the *Megavirales* representatives (Figure 4). *P. sibericum* was part of this monophyletic viral clade. Nevertheless, *P. sibericum* was not clustered with any other viral families or putative families. Finally, phylogenetic analysis performed based on the ATP-dependent DNA ligase, which is not conserved in the *Iridoviridae, Ascoviridae*, and *Mimiviridae* families apart from some distant representatives, showed a complex evolutionary history. In this phylogenetic reconstruction, megaviruses appeared as a paraphyletic clade scattered in two groups (Supplementary Figure S1). *P. sibericum* was clustered deeply with the *Marseilleviridae* and *Asfarviridae* families, which supports its evolutionary relationship with *Megavirales*; bacteriophages formed a sister group of these viruses.

### Phyletic analysis

Hierarchical clustering analysis was performed based on a binary presence**/**absence matrix constructed using 727 informational COGs that were known to be conserved in all cellular organisms among *Bacteria, Archaea*, and *Eukarya*, and some *Megavirales* members.

**Figure 5:**
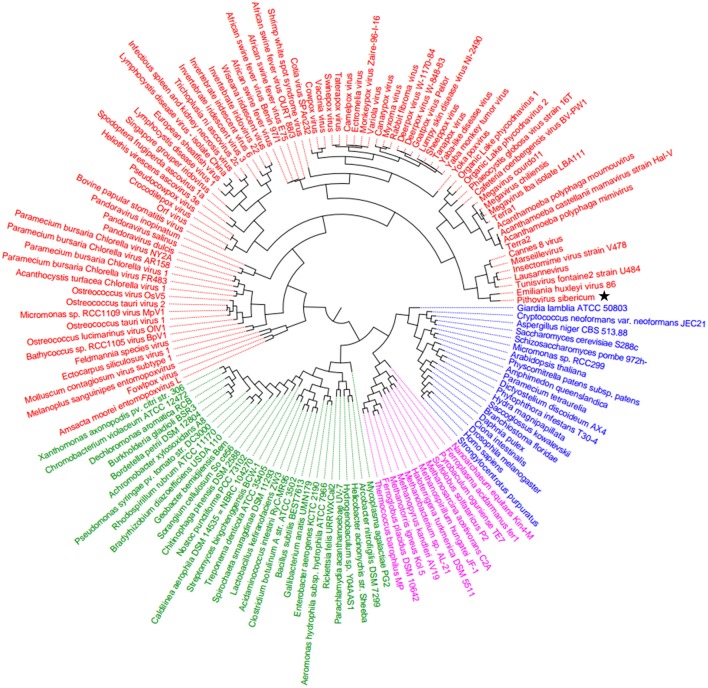
**Hierarchical clustering dendrogram**. The dendrogram tree was generated based on the presence/absence of genes in a matrix of informational COGs from *Megavirales* (red), *Bacteria* (green), *Archaea* (pink), and *Eukarya* (blue). The star indicates *Pithovirus sibericum*.

The corresponding binary matrix was composed of 142 representatives from *Bacteria, Archaea, Eukarya*, and *Megavirales*. The *P. sibericum* genome was found to encode homologs to a total of 19 COGs. Among them, the largest numbers of COGs were shared with representatives from the *Marseilleviridae* and *Mimiviridae* families (13 and 10, respectively). In addition, five COGs were shared with another *Megavirales* member, and only one of the 19 COGs present in *P. sibericum* was absent from any other *Megavirales* representatives. A total of 74 COGs were absent from *P. sibericum* while present in other *Megavirales* representatives, eight being present in Marseillevirus, and 23 being present in Mimivirus. Finally, this phyletic analysis based on a set of informational genes also showed a topology of four branches with *Megavirales* being a distinct and separate branch alongside *Bacteria, Archaea*, and *Eukarya*, and supports *P. sibericum's* belonging to the fourth TRUC (Figure 5).

## Discussion

The present phylogenetic and phyletic analyses including *P. sibericum* suggests that this giant virus is linked to *Megavirales*, which comprises a monophyletic group alongside *Eukarya, Bacteria*, and *Archaea*. Thus, phylogenies constructed based on three ancient genes (RNAP1/2, DNA polymerase, and TFIIB) strongly support *Megavirales* as an independent monophylogenetic group, as previously reported (Boyer et al., 2010; Sharma et al., 2014, 2015). Moreover, these analyses suggest that *P. sibericum* is the founder of a new viral family most closely related to the *Marseilleviridae, Iridoviridae*, and *Ascoviridae* families. *P. sibericum* position in both RNAP1 and RNAP2 trees indicated that this species belongs to a new family of *Megavirales*, and was most closely related to families *Iridoviridae/Ascoviridae* and *Marseilleviridae*, in congruence with previous analyses (Legendre et al., 2014; Yutin et al., 2014). Phylogeny reconstruction based on DNApol showed that *P. sibericum* branched deeply in a cluster that also included marseilleviruses, iridoviruses and ascoviruses, in congruence with a previous report (Legendre et al., 2014). Monophyly observed here for *Megavirales* members based on DNApol gene was not found in a previous study (Yutin and Koonin, 2012), whereas the one found based on the TFIIB gene was already reported (Yutin and Koonin, 2012). Otherwise, in accordance with the results from our phylogenetic analyses, hierarchical clustering performed here using a set of informational COGs showed that *P. sibericum* is a member of the order *Megavirales*, which comprises, as previously described, an independent branch, a fourth TRUC alongside cellular branches of life (Boyer et al., 2010; Raoult, 2013).

It was described initially based on comparative genomics and cladistic analyses that four viral families comprised a monophyletic group and shared an ancient common ancestor (Iyer et al., 2001; Koonin and Yutin, 2010). Later on, mimiviruses and additional new giant viruses have grown this monophyletic group and founded new families or putative families, which were proposed to be reclassified in the new order *Megavirales* (Colson et al., 2013; Sharma et al., 2015). The order *Megavirales* is a matter of debate because giant virus genomes comprise numerous genes that have orthologs in cellular organisms but a limited set of viral core genes (Yutin et al., 2014). Phylogenomic analyses using multiple core genes revealed complex evolutionary histories, and it was suggested that *Megavirales* members were gene robbers, and had acquired multiple genes from other megaviruses or from cellular organisms (Moreira and Brochier-Armanet, 2008; Williams et al., 2011; Yutin and Koonin, 2012). Overall, using different components of such chimeric gene repertoires as those of *Megavirales* representatives leads to different scenario based on the genes used. It has been put forward that part of the *Megavirales* genes have a cellular origin, but notwithstanding, a viral origin has been inferred for other genes in the genomes of these viruses, which have no detectable homologs in cellular life forms. Ancient viral genes with cellular counterparts also exist, and these latter show *Megavirales* as gathered biological entities apart from *Bacteria, Archaea*, and *Eukarya* (Boyer et al., 2010; Colson et al., 2011; Sharma et al., 2014, 2015).

Some previous studies have stated that *Megavirales* does not comprise a separate clade and should not be included in the tree of life, and are opposed to the fourth TRUC hypothesis (Moreira and Brochier-Armanet, 2008; Williams et al., 2011; Yutin et al., 2014). Notably, it was assumed that even ancient genes such as RNAP have a polyphyletic origin (Yutin et al., 2014). Thus, a polyphyletic origin of RNAP1/2 from *Megavirales* representatives was previously described, but in these studies the three eukaryotic paralogous genes were included whereas here we used RNA polymerase III, because it was found to be the most conserved of these three eukaryotic paralogs among *Megavirales* members and cellular living forms (Yutin and Koonin, 2012; Sharma et al., 2014, 2015; Yutin et al., 2014). In addition, RNAP1/2 genes from *Phytophtora parasitica*, a eukaryotic plant pathogen from the *Oomycetes* class, were found to be clustered with *Mimiviridae* (Yutin et al., 2014), but *Phytophtora parasitica* sequences were previously described to have *Megavirales* member sequences as best hits, and might represent overlooked megaviruses (Sharma et al., 2014). Misannotations exist in public databases, and contigs composed of viral genes have been detected within eukaryotic genomes (Filee, 2014; Maumus et al., 2014; Sharma et al., 2014). This might be explained by contamination with viral DNA, the sequencing of giant virus DNA together with that of a eukaryotic host, or the presence of giant viral genes in eukaryotic DNA. In any case, phylogenetic analyses using RNAP genes allowed us to resolve the phylogenetic and taxonomic status of megaviruses (Sharma et al., 2014). Certainly, giant viruses are chimeric organisms that harbor genes with multiple origins and a complex evolutionary history (Filee et al., 2008; Yutin and Koonin, 2012). However, there are still a few genes that are not prone to transfer, including those encoding DNA-dependent RNA polymerase subunits, or other informational genes, which enabled a better understanding of the origin and evolution of the giant viruses (Sharma et al., 2014). We believe that the *Megavirales* ancestor would have harbored these ancient stigma genes that exhibit few or no gene transfers. Then, *Megavirales* representatives might have lost some of these genes and acquired new ones from their respective hosts or from various organisms. Interestingly, previous analyses indicated that *P. sibericum* dramatically diverged from other viruses, similarly to pandoraviruses. Thus, *P. sibericum* shares only a small fraction of its genes with other known members of the *Megavirales* as only one third of its gene repertoire matches known viral genomes, and notably this repertoire lacks many of the megaviral core genes (Legendre et al., 2014). Nonetheless, the small core gene repertoire of this giant virus contains stigma genes that are sufficient to link it to the *Megavirales*.

Taken together, our phylogenetic and phyletic analyses indicate that *P. sibericum* is a new genuine member of the “Fourth TRUC” club, and appears to be closely related to the *Marseilleviridae* and *Iridoviridae/Ascoviridae* families.

### Conflict of interest statement

The authors declare that the research was conducted in the absence of any commercial or financial relationships that could be construed as a potential conflict of interest.

## Supplementary material

Supplementary Figure S1 **ATP-dependent DNA ligase phylogenetic tree**. The DNA ligase tree was built by using aligned protein sequences from *Megavirales* (red), *Bacteria* (green), *Archaea* (pink), and *Eukarya* (blue). Confidence values were calculated by the SH support using the FastTree program (Price et al., 2010). The scale bar represents the number of estimated changes per position. The star indicates *Pithovirus sibericum*.
