# Supplementary figures and images for: Pithovirus sibericum, a new bona fide member of the “Fourth TRUC” club
Source: Front Microbiol. 2015 Aug 4;6:722. doi: 10.3389/fmicb.2015.00722 (PMC4523831; doi:10.3389/fmicb.2015.00722)

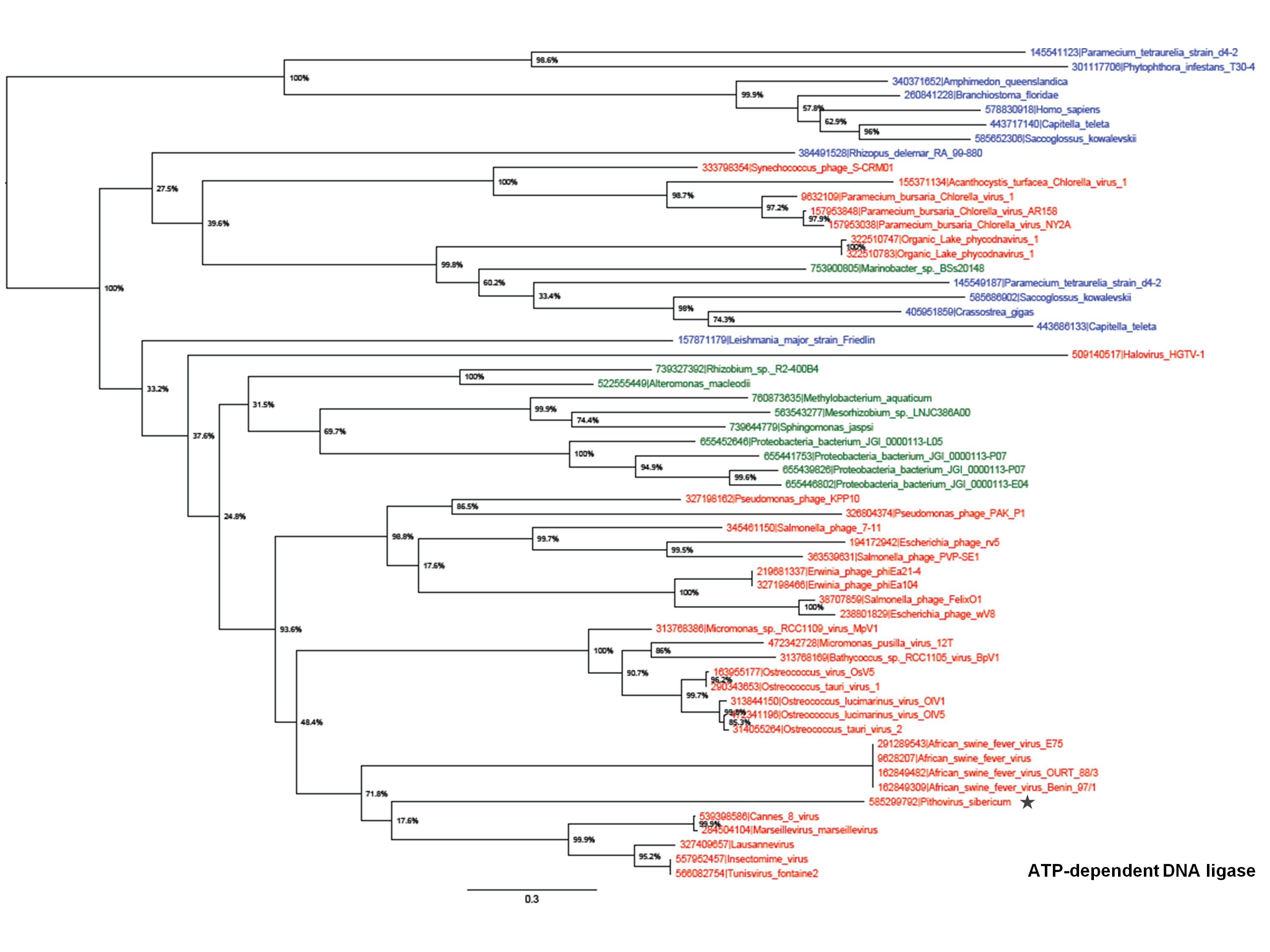

Supplement: Supplementary Figure S1 — ATP-dependent DNA ligase phylogenetic tree. The DNA ligase tree was built by using aligned protein sequences from Megavirales (red), Bacteria (green), Archaea (pink), and Eukarya (blue). Confidence values were calculated by the SH support using the FastTree program (Price et al., 2010). The scale bar represents the number of estimated changes per position. The star indicates Pithovirus sibericum. [file Image1.TIF]
